# Novel hybrid polyester-polyacrylate hydrogels enriched with platelet-derived growth factor for chondrogenic differentiation of adipose-derived mesenchymal stem cells in vitro

**DOI:** 10.1186/s13036-021-00257-6

**Published:** 2021-02-15

**Authors:** Fereshteh Valipour, Farzaneh Valipour, Reza Rahbarghazi, Amir Mohammad Navali, Mohammad Reza Rashidi, Soodabeh Davaran

**Affiliations:** 1 Stem Cell Research Center, Tabriz University of Medical Sciences, Tabriz, Iran; 2 Department of Medicinal Chemistry, Faculty of Pharmacy, Tabriz University of Medical Sciences, Tabriz, Iran; 3 Department of Molecular Biology, Faculty of Science, Hacettepe University, Ankara, Turkey; 4 Department of Applied Cell Sciences, Faculty of Advanced Medical Sciences, Tabriz University of Medical Sciences, Tabriz, Iran; 5 Department of Orthopedy, Tabriz University of Medical Sciences, Tabriz, Iran; 6 Applied Drug Research Center, Tabriz University of Medical Sciences, Tabriz, Iran

**Keywords:** Nanostructured scaffold, Thermosensitive hydrogel, Adipose-derived stem cells, Chondrogenesis

## Abstract

**Background:**

The goal of the present study was to create a new biodegradable hybrid PCL-P (HEMA-NIPAAm) thermosensitive hydrogel scaffold by grafting PNIPAAm-based copolymers with biodegradable polyesters to promote the chondrogenic differentiation of human progenitor cells (adipose-derived stem cells-hASCs) in the presence of the platelet-derived growth factor (PDGF-BB). Different mixture ratios including 50 mmol ε-caprolactone and 10 mmol HEMA (S-1), 30 mmol ε-caprolactone and 10 mmol HEMA (S-2), 10 mmol ε-caprolactone and 30 mmol HEMA (S-3) were copolymerized followed by the addition of NIPAAm.

**Results:**

A mild to moderate swelling and wettability rates were found in S-2 group copmpared to the S-1 ans S-3 samples. After 7 weeks, S-2 degradation rate reached ~ 43.78%. According to the LCST values, S-2, reaching 37 °C, was selected for different in vitro assays. SEM imaging showed nanoparticulate structure of the scaffold with particle size dimensions of about 62–85 nm. Compressive strength, Young’s modulus, and compressive strain (%) of S-2 were 44.8 MPa, 0.7 MPa, and 75.5%. An evaluation of total proteins showed that the scaffold had the potential to gradually release PDGF-BB. When hASCs were cultured on PCL-P (HEMA-NIPAAm) in the presence of PDGF-BB, the cells effectively attached and flattened on the scaffold surface for a period of at least 14 days, the longest time point evaluated, with increased cell viability rates as measured by performing an MTT assay (*p* < 0.05). Finally, a real-time RT-PCR analysis demonstrated that the combination of PCL-P (HEMA-NIPAAm) and PDGF-BB promoted the chondrogenesis of hASCs over a period of 14 days by up-regulating the expression of aggrecan, type-II collagen, SOX9, and integrin β1 compared with the non-treated control group (*p* < 0.05).

**Conclusion:**

These results demonstrate that the PCL-P(HEMA-NIPAAm) hydrogel scaffold carrying PDGF-BB as a matrix for hASC cell seeding is a valuable system that may be used in the future as a three-dimensional construct for implantation in cartilage injuries.

## Background

Articular cartilage, an avascular supporting connective tissue, is composed of extracellular matrix (ECM) and functional chondrocytes [1–3]. Due to the lack of sufficient blood vessels and low cell density, the cartilage has a limited ability to self-repair and regeneration owing to its lack of blood vessels and low cell density [4]. Conventional available therapies for cartilage diseases and injuries do not completely regenerate the injured sites, leading to the formation of fibrous masses [5, 6].

To circumvent these pitfalls and limitations, cartilage tissue engineering is a valuable concept to enhance the repair of cartilage defects and injuries [7]. In this approach, the combination of cells, scaffolds, and growth factors may improve the functionality of cells after transplantation to the target sites [8, 9]. Yet, in light of the limited number of tissue donors and of chondrocytes available in the cartilage for explantation, the de-differentiation potential of these cells, and possible rejection and/or transmission of infection issues after implantation, many attempts have been developed to identify other, more reliable sources of transplantable cells [10, 11]. Among them, mesenchymal stem cells (MSCs) were reported to exhibit a reliable chondrogenic differentiation potential, being also capable of surviving in long-term transplantation settings inside a target tissue [12]. Equally important, MSCs display immunomodulatory activities that make them a suitable allogeneic cell source for transplantation [13]. The adipose tissue is a particularly attractive source of MSCs, as it is conveniently accessible compared with other tissues such as bone marrow, synovium, dermis, peripheral blood, umbilical cord, and placenta [14, 15].

Scaffolds are critical components to promote the regeneration of cartilage injuries. These structures provide a three-dimensional (3D) environment comparable to the natural cartilage ECM. Compared with the conventional two-dimensional (2D) culture system, incubating cells in a 3D milieu provides essential cues for cell attachment and flattening, proliferation, migration, and differentiation while allowing for the synthesis of cell-derived ECM [16–18]. According to previous studies, a variety of natural and synthetic polymers have been used to fabricate scaffolds to support the chondrogenic differentiation of MSCs [19, 20]. Due to the existence of porous and water-swollen polymeric networks, hydrogels became the center of attention to generate scaffolds for many tissues and conditions [21], having the ability to initiate MSC chondrogenesis and supporting homogeneous cell seeding during encapsulation [4, 22, 23]. Poly N-isopropylacrylamide (PNIPAAm) hydrogels have been widely investigated as thermosensitive materials with lower critical solution temperature (LCST) at around 32 °C [24]. The LCST of the hydrogel can be modulated by copolymerization of NIPAM with hydrophilic or hydrophobic monomers to increase or decrease the transition temperature, respectively [25]. Poly (ε-caprolactone) (PCL) is a semicrystalline linear aliphatic polyester which combines several critical properties such as its biodegradability, biocompatibility, and good mechanical properties [26]. PCL can be degraded by hydrolysis of the ester bond in physiological conditions [27]. Unlike other polymers, PCL has less acidic degradation products and nontoxic nature, making it more suitable for implants, drug delivery, and tissue engineering applications [28, 29]. Of note, the hydrophobicity of PLC can be diminished by incorporation with other hydrophilic polymers [30]. Poly (2-hydroxyethyl methacrylate) (PHEMA) hydrogels, for example, are appropriate scaffold materials because of their biocompatibility, hydrophilicity, softness, high water content, and permeability. To fabricate amphiphilic materials, PHEMA hydrogels are being combined with hydrophobic components to improve mechanical strength [31].

In the present study, we aimed at fabricating a novel biodegradable and thermosensitive hybrid hydrogel composed of a PCL-P(HEMA-NIPAAm) hybrid copolymer. The chondrogenic capacity of human adipose-derived MSCs (hASCs) was investigated in the presence of platelet-derived growth factor (PDGF-BB) after being seeded on PCL-P(HEMA-NIPAAm). The results showed that PCL-P(HEMA-NIPAAm) is eligible to be used as a supportive scaffold to promote the differentiation of hASCs by providing certain physio-chemical properties. The culture of hASCs in the presence of PDGFR-BB increased the chondrogenic potential of PCL-P(HEMA-NIPAAm).

## Materials and methods

### Materials

The epsilon-caprolactone monomer (ε-CL; *99*% v/v) and 2*-*hydroxyethyl methacrylate (HEMA; 97% v/v) were purchased at Acros Organics (NJ, USA). HEMA was purified by vacuum distillation. N-isopropyl acrylamide (NIPAAm) was obtained at Acros Organics and purified by recrystallization from n-hexane-toluene (90:10 v/v). 92.5–100% Tin (II) 2-ethyl hexanoate (stannous octoate, Sn(Oct)_2_), benzoyl peroxide (BPO), 3,6-dimethyl-1 4-dioxane-2 5-dione, and 25% glutaraldehyde (aqueous solution) were from Sigma-Aldrich (Steinem, Germany). Dichloromethane (DCM, 99.5% v/v), ethanol, dimethyl sulfoxide (DMSO), n-hexane, toluene, chloroform, and isopropyl alcohol were obtained at Merck Chemical (Darmstadt, Germany). Phosphate buffered saline (PBS) and 0.25% trypsin/EDTA were from Gibco (Thermo Fisher Scientific, Inc.; Waltham, MA, USA). Low-glucose content Dulbecco’s modified Eagle medium (DMEM/LG) was obtained from Biowest (Nuaille, France). 3-(4,5-dimethylthiazol-2-yl-2,5-diphenyltetrazolium bromide) (MTT) and the penicillin-streptomycin (pen-strep) solution were purchased at Invitrogen Life Sciences (Nottingham, UK). Fetal bovine serum (FBS) was obtained from PAN Biotechnology (Aidenbach, Germany). Recombinant Human PDGF-BB peptide CF was purchased at R&D Systems (Minneapolis, MN, USA). The anti-CD133 (13A4), anti-CD44 (IM7), anti-CD34 (RAM34), and anti-CD45 (F10–89-4) antibodies were from eBioscience (San Diego, USA). PE- and FITC-conjugated secondary antibodies were from the same company (San Diego, USA). The SMART™ BCA (Bicinchoninic Acid) Protein Assay Kit was purchased at Intron Biotechnology (Sungnam, Korea). The TRIzol™ reagent was purchased from Yekta Tajhiz Company (Tehran, Iran).

### Synthesis of the HEMA-PCL macromonomer

**Fig. 1:**
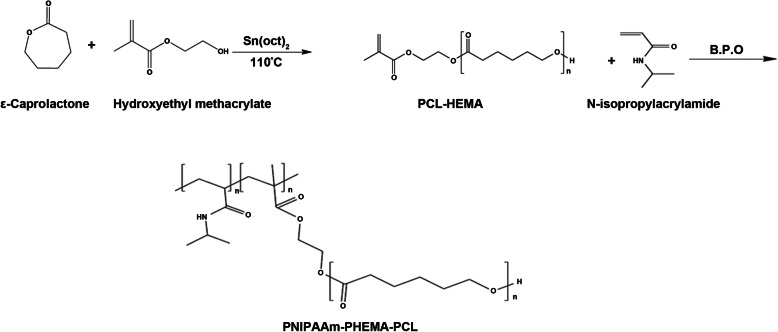
Synthesis scheme of the HEMA-PCL macromonomer and PCL-P(HEMA-NIPAAm) hybrid copolymer

The HEMA-PCL macromonomer was synthesized by ring-opening polymerization of ε-caprolactone in the presence of HEMA as an initiator and stannous octoate as a catalyst without solvent in bulk conditions [32] (Fig. 1). Different mixture ratios including 5.707 g (50 mmol) ε-caprolactone and 1.3 g (10 mmol) HEMA (S-1), 3.425 g (30 mmol) ε-caprolactone and 1.3 g (10 mmol) HEMA (S-2), 1.142 g (10 mmol) ε-caprolactone and 3.9 g (30 mmol) HEMA (S-3) were stirred individually at 110 °C in a nitrogen atmosphere for 30 min. Subsequently, a catalytic amount of Sn(Oct)_2_ (with an initial weight ratio of 0.05 wt% of the total monomer amount) was dissolved in about 0.5 ml of anhydrous toluene and added to the mixture. The reaction was carried out at 110 °C for 4 h under nitrogen atmosphere in a stirred flask, and the temperature was precisely controlled by an external oil bath. All the macromonomers produced were refrigerated at 4 °C until use. After cooling to RT, the resulting product was dissolved in CHCl_3_. The solution was added dropwise into the excess ethanol to precipitate. The obtained macromonomer was dried in a vacuum at RT. The structure of the macromonomer was characterized by Fourier-transform infrared spectroscopy (FT-IR).

### Synthesis of the PCL-P(HEMA-NIPAAm) hybrid copolymer

PCL-P(HEMA-NIPAAm) hybrid copolymers were synthesized by free-radical copolymerization of NIPAAm (10 g) and HEMA-PCL macromonomers (3.33 g) in 1,4-dioxane (50 ml) under nitrogen atmosphere (Fig. 1). The mixture was degassed under nitrogen atmosphere for 30 min before polymerization. BPO at a concentration of 0.05 wt% of the total weight of macromonomer and NIPAAm was used as a copolymerization initiator. The polymerization was carried out at 70 °C for 24 h under a nitrogen atmosphere [33, 34]. After reaching RT, the product was purified by dissolution/precipitation with dichloromethane/ethanol solution and then dried in a vacuum at RT. The purified product was lyophilized and stored at 4 °C until use. The structure of the cross-linked hydrogel was characterized by FT-IR and ^1^H-NMR.

### System characterization methods

FT-IR spectra of the synthesized HEMA-PCL macromonomer and PCL-P(HEMA-NIPAAm) hybrid copolymer were obtained in the range of 4000 to 400 cm^− 1^ using an FT-IR spectrometer (Tensor 27 spectrophotometer, Bruker; Avance, Germany). The samples were ground and mixed thoroughly with potassium bromide. The final concentration of each sample in KBr was 1%. The chemical composition of the PCL-P(HEMA-NIPAAm) hybrid copolymer was analyzed by hydrogen nuclear magnetic resonance (^1^HNMR) spectroscopy at 400 MHz using a Bruker spectrometer (Ettlingen, Germany) and deuterated chloroform (CDCl_3_) as a solvent. To investigate the wettability and hydrophobicity of scaffolds, water contact angles test was performed. Static water contact angles of scaffolds were carried out through the sessile drop method by a goniometer (Model no. CA-500A; Sharifsazan; Tabriz, Iran) at room temperature. For this purpose, the scaffolds were cut into square specimens with the size of 1 cm × 1 cm. Subsequently, 2 μl distilled water was dropped onto the each scaffold surface. The water contact angle was obtained after 10 s and the drop dimension parameters were automatically calculated from the digitalized image. The measurements recorded were an average of at least five contact angles obtained on three samples. The experiments were performed at least in triplicate. The rate of water absorption of the synthesized porous scaffolds was measured by the gravimetric method in PBS with pH = 7.4 at physiological temperature (37 °C). The dried scaffolds were cut into similar size particles and weighted before incubation (W_d_). Scaffolds were immersed in PBS buffer solution at predetermined times. The scaffolds were taken out and their wet weight was determined (W_w_) by the exclusion of excess surface water using filter paper [35]. The ratio of swelling was calculated using eq. 1:

1 $$ \mathrm{swelling}\ \mathrm{ration}\kern0.5em =\kern0.5em \left({\mathrm{W}}_{\mathrm{w}}\hbox{-} {\mathrm{W}}_{\mathrm{d}}\right)/{\mathrm{W}}_{\mathrm{d}} $$

All experiments were performed triplicate and the average values have been reported in the scheme. To evaluate the in vitro degradation of synthesized hydrogels, all the samples were lyophilized and weighed before (W_0_) and after (W_t_) soaking in PBS with pH = 7.4 at 37 °C in respective times [36]. The samples were then removed from the solution, vacuum-dried at RT to reach a constant weight, and weighed again. The hydrogel degradation rate was measured by monitoring the weight loss over time using eq. 2:

2 $$ \%\mathrm{weight}\ \mathrm{loss}\kern0.5em =\kern0.5em \left[\left({\mathrm{W}}_0\hbox{-} {\mathrm{W}}_{\mathrm{t}}\right)/{\mathrm{W}}_0\right]\kern0.5em \times \kern0.5em 100\% $$

where W_t_ is the initial dry weight of the sample and W_0_ is the dry weight of the sample after a specific degradation period. The culture medium was replenished every 3–4 days. Three sets of experiments were performed. To determine the LCST of PCL-P(HEMA-NIPAAm) hybrid hydrogels, the cloud point (CP) measurement (turbidimetry) method was used. Optical transmittance of aqueous polymer solution at various temperatures was measured at the wavelength of 500 nm using a UV-Vis spectrometer (UV-160 Shimadzu) with increasing solution temperatures (18–50 °C). The heating rate was 1 °C/min. At each step, the samples were stabilized for 10 min before the next measurements [33]. Values for the LCST of polymeric solutions were determined as the temperature at the inflection point in the normalized absorbance versus the temperature curve.

### Mechanical evaluations

The mechanical properties of the scaffold were calculated using the universal material testing machine STM250 (Santam; Tehran, Iran). Hydrogel samples were equilibrated for 3 days at 37 °C in PBS before testing. Hydrogel samples were cut into cubes (5 × 5 × 5.74 mm). The sample was placed on the sample plate of the compression tester. To study mechanical properties, the scaffolds were mechanically analyzed in compression using a 5 N load cell with a loading rate of 2 mm/min [37]. Using values obtained from the force and compression analyses, the stress-strain curves were plotted followed by calculation of Young’s modulus index.

### Scanning electron microscopy (SEM)

Morphological studies of the fabricated scaffold before and after cell culture were investigated using field emission scanning electron microscopy FESEM (JEOL, JSM 6700F model) (JEOL Ltd.; Tokyo, JAPAN). hASCs were seeded on the *freeze-dried* scaffold at a density of 5 × 10^4^ cells/cm^2^ and cultured 7 days with medium replenished every 2–3 days. After reaching appropriate confluency, scaffolds harboring hASCs were washed twice with PBS, fixed in 2.5% glutaraldehyde solution at RT for 2 h, rinsed three times with PBS, and dehydrated at the increasing percentage of ethanol (25–100%) for 20 min [38]. The samples were then dried overnight at RT and finally prepared for SEM utilizing a gold coating method and displayed at 15 kV.

### Serum protein adsorption capacity

To assess the possible capacity of the fabricated scaffold to adsorb different protein types, we measured the total content after incubation of scaffold with serum. For this purpose, scaffolds were cut in equal size and placed in 96-well plate, suspended in a 200 μl culture medium (DMEM/LG containing 10% FBS) and maintained inside an incubator at 37 °C for 2 weeks. Thereafter, the supernatant was collected and stored in microtubes. Then, the scaffolds were chopped into small pieces and 1 ml of *serum-free media* was added to it. The media was removed from the scaffolds and centrifuged at 14,000 rpm for 10 min to yield homogeneous liquid. The media was then collected again in another microtube to measure the levels of adsorbed protein. In the current experiment, we measured the protein concentration using the SMART™ BCA (Bicinchoninic Acid) Protein Assay Kit according to the manufacturer’s instructions. In short, 25 μl of each standard (bovine serum albumin - BSA), control, and protein samples were pipetted into a 96-well plate, and 150 μl of working solution (WS) to each well and mixed plate thoroughly on a plate shaker for 30 s. Then, the plates were covered and incubated at 37 °C for 2 h. The plate cooled to RT. Serum-free medium was used as blank. The absorbance of each well was at 570 nm using a microplate reader (BioTek; VT, USA). The experiments were performed in triplicate.

### Isolation and expansion of hASCs

To isolate hASCs, six normal women, ranging from 45 to 48 years old, were enrolled in the current experiment who referred to liposuction surgery at the Imam Reza Hospital, an affiliated hospital to Tabriz University of Medical Sciences (Tabriz, Iran). All procedures of the study were approved by the local ethics committee of Tabriz University of Medical Sciences (IR.TBZMED.REC.1395.74). To obtain samples, all volunteers were asked to complete and signed the informed consent forms. The samples were kept in PBS solution enriched with 1% Pen-Strep solution and transferred quickly to the Stem Cell Research Center, Tabriz University of Medical Sciences. Then, samples were carefully chopped into small size and incubated with an enzymatic solution containing 1% type 1 collagenase plus 0.25% Trypsin/EDTA at 37 °C for 30–40 min. The samples were continuously shaken to facilitate the release of cells from the tissue mass. Finally, the suspensions containing enzymes were blocked using FBS. Samples were centrifuged at 1000–2000 rpm and the cell pellet was resuspended in DMEM/LG supplemented with 10% FBS and 1% pen-strep. The cells were maintained in an incubator at 37 °C and a humidified atmosphere with 5% CO_2_. The supernatant medium was replaced every 2–3 days until 70–80% confluence. 0.25% trypsin/EDTA was used to detach and passaged the hASCs. Cells (passages 3–6) were employed in the experiments using the following groups: [hASCs], [hASCs + PDGF-BB], [hASCs + scaffold], and [hASCs + scaffold + PDGF-BB] using 10 ng/ml of PDGF-BB.

### Flow cytometry

hASCs were characterized for the expression of CD133, CD44, CD34, and CD45 by flow cytometric analysis. Cells at passage 3 were detached using 0.25% trypsin/EDTA, incubated with 1% BSA, and permeabilized with 0.1% triton-X100. After three washes with PBS, the cells were incubated with PE- and FITC-conjugated secondary antibodies at 4 °C for 30 min according to the manufacturer’s recommendations. 1 × 10^5^ cells were analyzed using BD FACSCalibur and FlowJo software (version 7.6.1) (TreeStar, Inc.; Ashland, OR, USA).

### Cytotoxic assay

The possible cytotoxicity of the synthesized scaffold on hASCs was assessed using the MTT assay. The scaffolds were lyophilized and sterilized after exposure to UV light in a laminar flow hood. Scaffolds (10 mm × 10 mm × 6–10 mm) were placed in 96-well plates followed by the addition of 200 μl DMEM/LG containing 1% pen-strep and 10% FBS. Plates were temporarily incubated at 37 °C for 30 min. The medium was next removed and 150 μl DMEM/LG containing 10% FBS and 1% pen-strep with a density of 10^4^ hASCs/well were added to each well and kept for 14 days under conventional condition. After completion of incubation time, the medium was discarded and 50 μl MTT solution (5 mg/ml) was added to each well. The plates were maintained at 37 °C for 4 h. Thereafter, the supernatants were semi-replaced by 100 μl of DMSO, and the plates were gently shaken for 15 min to dissolve formazan crystals [39]. The homogenous blue colored supernatants were next transferred to the fresh 96-well plates. Absorbance values of each well were measured by absorbance reading at 570 nm in an ELISA reader (BioTek). The final ODs were measured and expressed as % of control hASCs and experiments were carried out in triplicate. hASCs seeded on standard plastic culture surfaces were used as controls.c

### Real-time RT-PCR analysis

To evaluate the chondrogenic potential of scaffold on hASCs, cells were plated in each well of 6-well plates for 14 days and the expression of aggrecan, type-II collagen, SOX9, and integrin β1 was measured by real-time RT-PCR analysis, with glyceraldehyde-3-phosphate dehydrogenase (GAPDH) serving as a housekeeping gene and internal control (Table 1). After completion of cell culture, the media was aspirated and the cells were lysed directly by adding 1 ml of TRIzol™ reagent. After several pipetting, the homogenized samples were incubated at 25 °C for 5 min to permit the complete dissociation of nucleoprotein complexes. Then, 0.2 ml of chloroform solution was added and sample tubes capped securely and tubes were shaken vigorously followed by centrifugation at 12,000 x g at 4 °C for 5–10 min. The mixture was separated into a lower yellow, phenol-chloroform phase, interphase, and a colorless upper aqueous phase. The aqueous phase was transferred to a fresh tube. RNA was precipitated from the aqueous phase by mixing with 0.5 ml of isopropyl alcohol. Finally, the concentration of isolated RNAs was calculated based on the OD^260/280 nm^ ratio measurement by NanoDrop (NanoDrop one c, Thermo Fisher Scientific; MA, USA). By using cDNA synthetase (Yekta Tajhiz; Tehran, Iran), RNAs were reverse-transcribed into cDNA. Real-time RT-PCR was performed using the YTA SYBR Green qPCR Master Mix 2X kit on a Rotor-Gene Q (Qiagen; Hilden, Germany) real-time instrument. Fold change expression of each sample, calculated by the 2^-ΔΔCT^ method. Each sample type was run in triplicate. hASCs seeded on standard plastic culture surfaces (without scaffold and PDGF-BB) were used as controls.

**Table 1:** Primers employed in the real-time RT-PCR analysis

Genes	Sequences	T_m_ (°C)
Aggrecan	Forward: 5′-CCATCTCTACACGCTACACCC-3′ Reverse: 5′-TTGTCTCCATAGCAGCCTTCC-3’	60 °C
Type-II collagen	Forward: 5’-CGTCTACCCCAATCCAGCAAA-3′ Reverse: 5′-AGCAGGCGTAGGAAGGTCAT-3’	60 °C
SOX9	Forward: 5’-CACACTCCTCCTCCGGCATGA-3′ Reverse: 5′-GCGGAAGTCGATAGGGGGCT-3’	63 °C
Integrin β_1_	Forward: 5’-GCGAGTGTGGTGTCTGTAAGT-3′ Reverse: 5′-CATTCCTGTGTGCATGTGTCTT-3’	60 °C
GAPDH	Forward: 5’-AGCCAAAAGGGTCATCATCTCT-3′ Reverse: 5′-AGTCCTTCCACGATACCAAAGT-3’	60 °C

### Statistical analysis

Data are expressed as mean ± standard deviation (SD). The statistical difference between groups was calculated using the One-ANOVA assay and Tukey post hoc analysis (GraphPad Software, San Diego, CA, USA), with *p* < 0.050 considered statistically significant (*p < 0.050; **p < 0.010; ***p < 0.001; ****p < 0.0001).

## Results and discussion

### Fourier-transform infrared spectroscopy (FT-IR) analysis

**Fig. 2:**
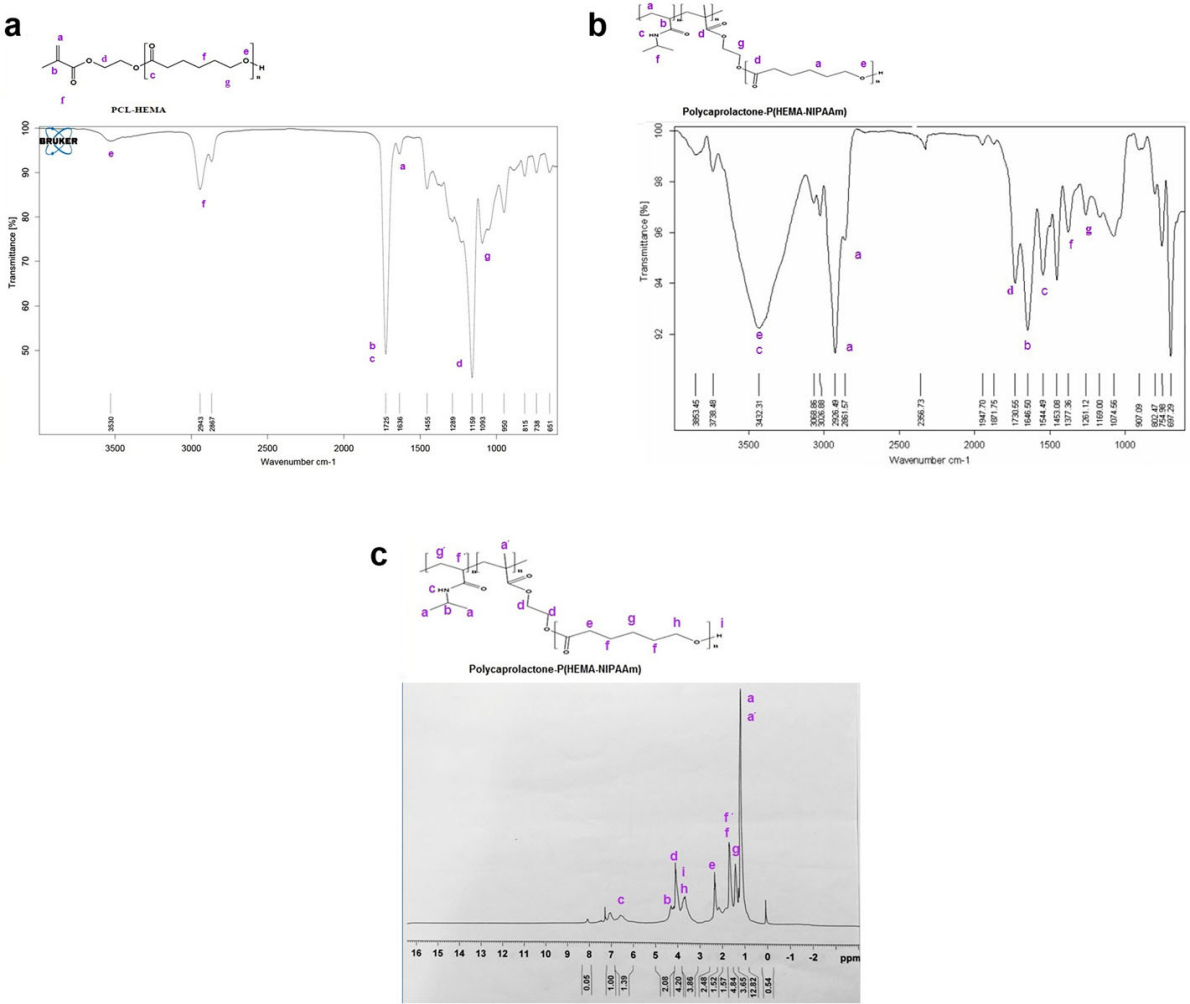
FT-IR spectra of the PCL-HEMA macromonomer (**a**). FT-IR spectra of the PCL-P(HEMA-NIPAAm) hybrid copolymer (**b**). ^1^HNMR spectrum of the PCL-P(HEMA-NIPAAm) hybrid copolymer (**c**)

FT-IR was first performed to investigate the chemical structure of the synthesized hybrid PNIPAAm-polyester scaffold. The FT-IR spectrum of the HEMA-PCL macromonomer and PCL-P(HEMA-NIPAAm) hybrid copolymer are presented in Fig. 2a and b, respectively. The spectrum of the HEMA-PCL macromonomer exhibited a characteristic stretching vibration peak for the ester carbonyl (C=O) at 1725 cm^− 1^ and a broad peak at 3530 cm^− 1^ for hydroxyl group (−OH). The bands at 2945 and 2820 cm^− 1^ are attributed to the stretching vibrations of (−CH_2_) and (−CH_3_). Moreover, there was an adsorption band at 1636 cm^− 1^ which is from the (C=C) stretching vibration of HEMA. The peak at 1159 cm^− 1^ is the stretching vibration absorption peak of (C-O-C) from HEMA and PCL. The peak at 1454 cm^− 1^ is assigned to the bending vibration of (−CH_2_-) groups. The PCL-P(HEMA-NIPAAm) copolymer (Fig. 2b) exhibited the characteristic absorption signals of amide functional groups of poly(NIPAAm) at 1646 cm^− 1^ (NH-CO- stretching), 1544 cm^− 1^ (N-H bending), and 1377 cm^− 1^ correspondings to the C-H vibrations of -CH(CH_3_)_2_ (41). The main absorption bands in this copolymer were the stretching vibrations of carbonyl groups of PHEMA, PCL at 1730 cm^− 1^, and PNIPAAm at 1652 cm^− 1^. The characteristic absorption band of the vinyl group at 1636 cm^− 1^ was not observed, indicating that the HEMA monomer was successfully converted to the PHEMA polymer through the free radical polymerization reaction [40]. The absorption bands due to (−NH secondary amid) and hydroxyl groups were overlapping and led to a very strong and broadband centered at 3456 cm^− 1^. The absorption at 2661 and 2926 cm^− 1^ corresponds to aliphatic -CH stretching vibrations of (−CH_3_) and (−CH_2_-) groups.

### Hydrogen nuclear magnetic resonance (^1^HNMR) spectrum

The ^1^HNMR spectrum (in CDCl_3_) of the PCL-P(HEMA-NIPAAm) copolymer was used to determine the structure and composition of the copolymer (Fig. 2c). The peak of methylene’s (CH_2_) in PCL was seen around 1.381 ppm (2H, g), 1.646 ppm (2H, f), 2.333 ppm (2H, e), and 3.685 ppm (2H, h). The signals at 6.56 ppm (H, c), 4.209 ppm (H, b), and 1.31 ppm (3H, a) correspond to protons of (NH-CH(CH_3_)_2_), (−CH(CH_3_)_2_) and methyl groups in the PNIPAAm segment. Overlapping resonance was related to methyl of the ester group (−OCCH_2_-) at 4.158 ppm (2H, d) in the PHEMA-PCL segment. The removal of the peaks at 5.58 ppm and 6.11 ppm indicates that end double bonds were converted into carbon-carbon signal bonds to form the main chain during polymerization [41]. All the evidence proved a graft copolymer was successfully synthesized [33, 42].

### Water contact angle measurement

**Fig. 3:**
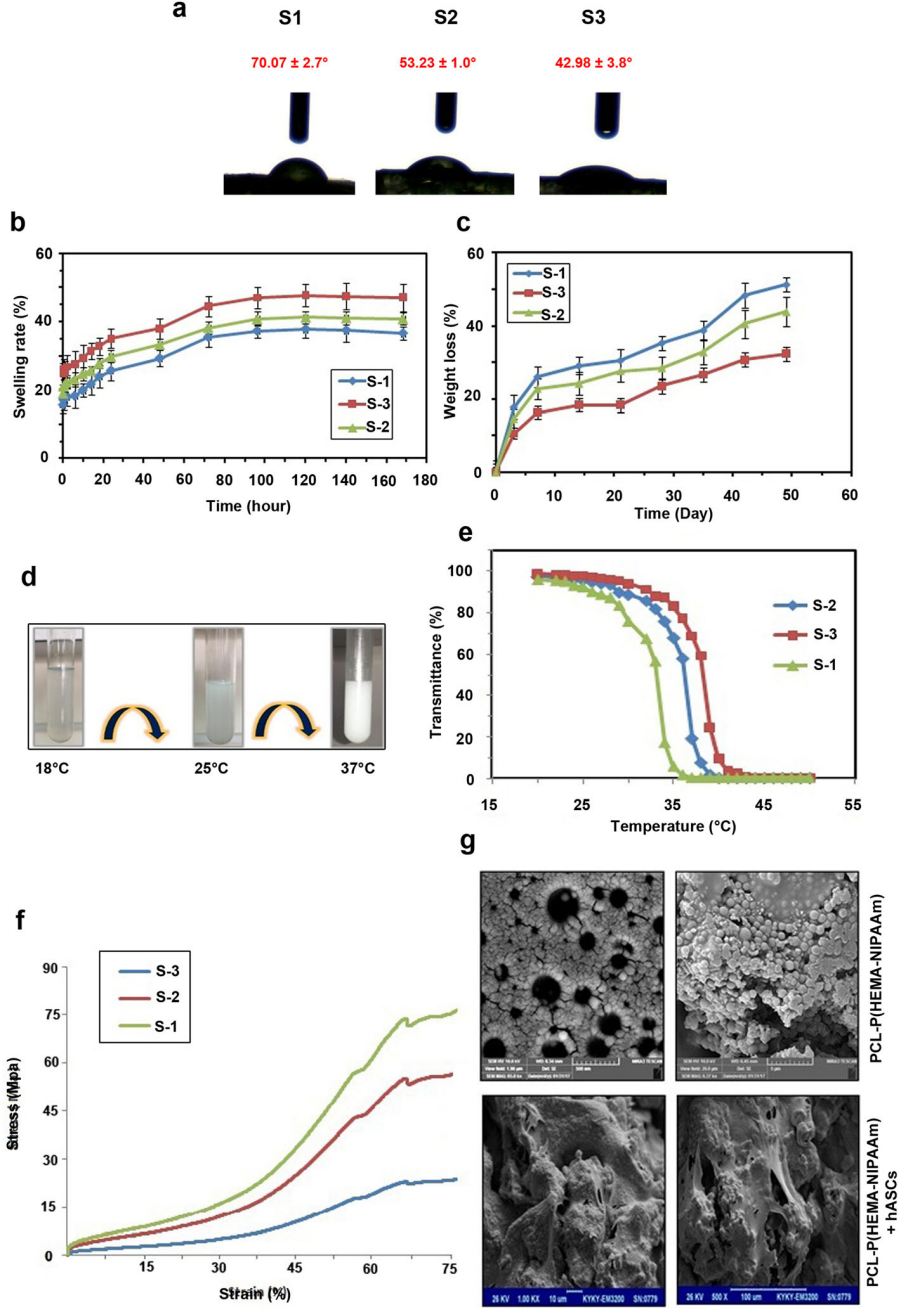
Measuring S-1, S-2, and S-3 water contact angle (**a**). Equilibrium swelling ratios and swelling profiles of the PCL-P(HEMA-NIPAAm) hydrogels with different concentrations of CL and HEMA (S-1, S-2, S-3) as a function of time at pH = 7.4 and 37 °C (*n* = 3) (**b**). In vitro hydrolytic degradation profiles of the PCL-P(HEMA-NIPAAm) hydrogels with different concentrations of CL and HEMA (S-1, S-2, S-3) as a function of time in PBS at 37 °C (*n* = 3) (**c**). Macroscopic phase separation of an aqueous solution of the PCL-P(HEMA-NIPAAm) S-2 hydrogel under different conditions (**d**). Transmittance-temperature curves of the PCL-P(HEMA-NIPAAm) hydrogels (**e**). Compressive stress-strain curve of the PCL-P(HEMA-NIPAAm) all hydrogels. Data showed that S-2 exhbits moderate compressive strength, Young’s modulus, and compressive strain (%) compared to the S-1 and S-3. FESEM micrographs of the architecture of the PCL-P(HEMA-NIPAAm) S-2 hydrogel surface (**g**). Observation of the cell morphology, attachment, and spreading of hASCs on the PCL-P(HEMA-NIPAAm) S-2 hydrogel after 7 days of culture through SEM

Wettability refers to the ability of scaffolds to adsorb water which is touted as primary concern both in vitro and in vivo culture systems [43]. Hydrophilic surfaces with small contact angles (less than 90°) corresponded to high wettability, while hydrophobic surfaces with large contact angles (more than 90°) corresponded to low wettability [43]. In this study, we evaluated the relative hydrophilicity of scaffolds by measuring their water contact angles using a sessile drop method (Fig. 3a). Data revealed that water contact angles of S-1, S-2 and S-3 scaffolds were 70.07 ± 2.7, 53.23 ± 1.0 and 42.98 ± 3.8 degrees. It has shown that the contact angle of the scaffolds has propotional correlation with the levels of PCL and reduction PHEMA in the final components. Therefore, we can changes waterabiltiy index by the alteration of PCL and P(HEMA). The enhancement in the wettability can improve the cell attachment, proliferation and mutual interaction of cells with the scaffolds [43]. Considering the efficiency of mild to moderate wettability index in the growth dynamics, we selected S-2 scaffold for subsequent assays.

### Swelling studies of the PCL-P(HEMA-NIPAAm) hydrogels

The swelling capability of the scaffold correlates with the cell infiltration into the scaffolds in a 3D culture system [44]. It also increases the surface area, allowing cells to adhere to the surface of the scaffold and the samples *to be* available *for* nutrients from culture media more effectively [45]. Excessive swelling may lead to an increased pore size while affecting the attachment and proliferation of cells. In contrast, a lower swelling degree could help to enhance scaffold mechanical stability [46]. Thus, a scaffold with a controlled swelling ratio is desired for the regeneration of different tissues. The equilibrium swelling ratios and swelling profile as a function of time for the PCL-P(HEMA-NIPAAm) hydrogels containing a fixed concentration of NIPAAM and different concentration of CL and HEMA are illustrated in Fig. 3b. All scaffolds had similar swelling behaviors as a function of time and showing controlled swelling behavior in the PBS medium. The swelling ratio increased with an increase in the hydrophilic HEMA content. According to the data, the addition of the hydrophobic PCL segment decreased the swelling rate. This result is supported by a study, which reported by Chao et al. [47]. In this study, the swelling ratio of S-3 was higher than that of S-2 and S-1 at pH = 7.4 and 37 °C.

### In vitro degradation of the PCL-P(HEMA-NIPAAm) hydrogels

The degradation ability of a hydrogel scaffold is a vital parameter for successful scaffold fabrication [45]. To achieve long-term mechanical stability and reliability, scaffolds should degrade slowly over time in a controlled manner. The hydrolysis of the hydrogels was studied in simulated physiological conditions in a buffer solution of pH 7.4 at 37 °C. As shown in Fig. 3c, the degradation rate was mainly determined by the biodegradability of the PCL segment. The hydrolytic degradation rate of the scaffolds was increased with the increasing content of PCL in the hydrogels. PCL segments were hydrolytic degradable, and weight loss of the hydrogels was mainly caused by cleavage of ester bonds within the network in PCL segments. This finding is consistent with the results reported by Chao et al. [47]. In conclusion, weight loss of the hydrogels increased with increase in PCL content or decrease in HEMA content. In the current experiment, the hydrogels exhibited a substantial weight loss, approximately 32.33% (S-1), 43.78% (S-2), and 51.24% (S-3) of its initial mass after 7 weeks. Concretely, the use of slowly degrading scaffolds gives the constructs evaluated in this study long-term stability, maintaining a biomechanically functional framework that supplies seeded cells substantial time to synthesize a new ECM.

### LCST and phase transition behavior of the PCL-P(HEMA-NIPAAm) hydrogels

LCST of the hydrogels was investigated by the turbidity method by monitoring the change in the optical absorbance as a function of temperature (Fig. 3d-e). The results confirmed a similar sharp phase transition for hydrogels with a small temperature change. The sharp reduction in UV transmittance indicates that the hydrogels become increasingly turbid with shrinking on increasing at higher temperatures, and the LCST is the point at which the network structure of polymer collapse and water expelled of the hydrogel. As indicated in Fig. 3d-e and Table 2, LCST values of the hydrogels were obtained with a constant concentration of NIPAAm and different concentrations of CL and HEMA at 34 °C, 37 °C, and 39 °C, respectively. The ability of hydrogels to absorb water arises from hydrophilic functional groups and *hydrogen bonding ability between polymer and water* increases, whereas the LCST of hydrogels decreased when increasing the hydrophobic PCL segment because the hydrophobic comonomer facilitates chain aggregation [48]. Figure 3d-e shows the visual macroscopic observation of phase separation of aqueous hydrogel solution below and above LCST. As illustrated in Fig. 3d-e, below the LCST the solution of the hydrogel is clear but upon heating, at 37 °C, the solution became turbid because of the aggregation of the hydrogel. It is important to note that the LSCT of the S-2 hydrogel is similar to the body temperature, a valuable feature for biological applications. According to these results, S-2 was selected for further biological assays.

**Table 2:** Composition and LCST of PCL-P(HEMA-NIPAAm) hybrid hydrogels

Groups	CL (g)	HEMA (g)	PCL-HEMA ratio	LCST (°C)
S-1	5.707	1.3	5:1	34
S-2	3.425	1.3	3:1	37
S-3	1.142	3.9	1:3	39

Final wt% of NIPAAm was equal in all groups (70% NIPAAm:30% PCL-HEMA). Abbreviations: *CL* Caprolactone, *HEMA 2-hydroxyethyl methacrylate*, *PCL* Poly (ε-caprolactone), *LCST* Lower critical solution temperature, *NIPAAm* N-isopropyl acrylamide

### Mechanical properties of the PCL-P(HEMA-NIPAAm) hydrogels

Compressive property is one of the most important mechanical characteristics of scaffolds that are especially vital for hydrogels used in tissue engineering [49]. Scaffolds should have sufficient mechanical strength to withstand mechanical stress to provide the appropriate biomechanical environment needed for cell migration, proliferation, and differentiation [37]. To this end, the scaffolds used in tissue engineering should have adequate structural stability to retain their initial shape at the implant site until their function gets completed [50]. To study the mechanical behavior of the prepared scaffolds, the mechanical strength of the scaffolds was investigated via the uniaxial compression technique. The typical stress-strain plot of the PCL-P(HEMA-NIPAAm) hydrogels is illustrated in Fig. 3f. As seen in the diagram, the stress-strain curve of three scaffolds exhibits three distinct regions: linear elastic behavior at low stress, non-linear shifting strain at intermediate stress, and linear elastic behavior at high stress. The modulus of elasticity (E) was calculated as the slope of the linear portion of the stress-strain curve. The compressive strength, Young’s modulus, and compressive strain (%) of the S-3 were highest while we achieved lowest values for S-1. The intermediate compressive strength, Young’s modulus, and compressive strain (%) were reported for the S-2 (44.8 MPa, 0.7 MPa, and 75.5%, respectively). The Young’s modulus of cartilage is in the range of 0.45–0.90 MPa [51]. Based on compressive modulus results, the PCL-P(HEMA-NIPAAm) hydrogel may be the most suitable candidate for cartilage engineering.

### SEM analysis of the PCL-P(HEMA-NIPAAm) S-2 hydrogel

The morphology of the fabricated freeze-dried S-2 scaffold was demonstrated by FESEM micrographs before and after cell culture (Fig. 3g). SEM micrographs were used to study the attachment, morphology, and spreading of cells on the scaffolds. According to Fig. 3g, the PCL-P(HEMA-NIPAAm) hydrogel had a nanoparticulate structure with particle size dimensions about 62–85 nm with uniform and highly interconnected porous structures. The minimum and maximum sizes of the pores ranged between 8 to 14 μm, respectively. By 7 days following hASCs seeding on the PCL-P(HEMA-NIPAAm) hydrogel (Fig. 3g), the cells adhered well and covered the surface of the scaffold. Attachment of hASCs to the surface of the PCL-P(HEMA-NIPAAm) hydrogel was associated with a marked morphological adaptation. In the conventional 2D culture system, hASCs acquired spindle-like appearance while 7-days culture on the PCL-P(HEMA-NIPAAm) hydrogel contributed to cell flattening. According to Fig. 3g, hASCs were shaped based on the surface properties of the scaffold. These images give visual evidence that hASCs could attach and undergo dramatic morphological changes. Also, effective cell connection to the scaffold surface indicates that the PCL-P(HEMA-NIPAAm) hydrogel is non-toxic for hASCs. The proximity of the cultured hASCs on the PCL-P(HEMA-NIPAAm) surface shows that the scaffold is suitable to maintain the cell-to-cell connection which is vital for the dynamic growth.

### Adsorption of serum protein on the PCL-P(HEMA-NIPAAm) S-2 hydrogel

**Fig. 4:**
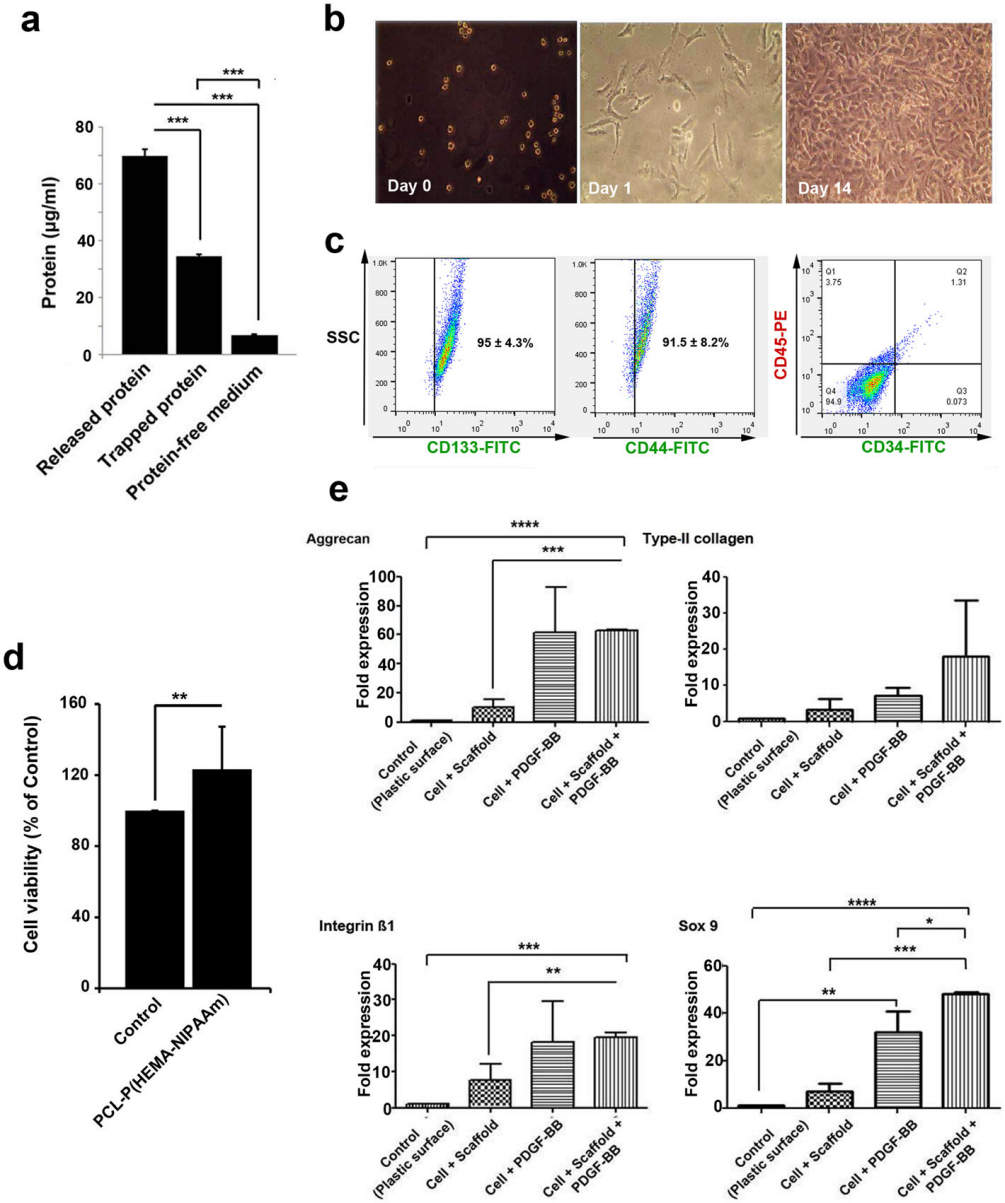
Measurements of the total protein contents in the PCL-P(HEMA-NIPAAm) S-2 hydrogel after 2 weeks of incubation with serum-containing medium using a BCA assay as described in the Materials and Methods. ***p < 0.001 (**a**). Morphological analysis by inverted phase-contrast microscopy (magnification × 100) at a different stages of culture and proliferation (days 0, 1, and 14) (**b**). Characterization of hASCs (**c**). Detection of hASC surface marker expression (CD133, CD44, CD34, and CD45) by flow cytometry as described in the Materials and Methods. Viability of hASCs in the PCL-P(HEMA-NIPAAm) S-2 hydrogel. Samples were processed using the cytotoxicity assay after 14 days. **p < 0.01 (**d**). Real-time RT-PCR analysis of the gene expression profiles in hASCs seeded on the PCL-P(HEMA-NIPAAm) S-2 hydrogel (**e**). Aggrecan, type-II collagen, SOX9, and integrin β1 expression was analyzed using the 2^-ΔΔCT^ method. GAPDH was used as a housekeeping gene and internal control. *p < 0.050, **p < 0.010, ***p < 0.001, and ****p < 0.0001

Protein adsorption onto the surface of materials is extremely related to the biocompatibility of the materials and used to evaluate the biological properties of the scaffolds [52]. For this purpose, the PCL-P(HEMA-NIPAAm) S-2 hydrogel was incubated in culture media containing serum protein for 14 days and the level of adsorbed protein assessed by BCA protein quantitative assay. The amount of total serum protein adsorption was significantly high on the hydrogel scaffold compared with serum-free culture medium (*p* < 0.001), showing that significant amounts of serum protein were adsorbed by the PCL-P(HEMA-NIPAAm) hydrogel. Despite these advantages, we found that the levels of released protein were higher compared with the trapped protein content (*p* < 0.001), showing the ability of the synthesized scaffold to release protein to the surrounding niche (Fig. 4a). These findings suggest that the PCL-P(HEMA-NIPAAm) hydrogel may have certain characteristics improving specific protein-affinity and binding strength. Further experiments are needed to determine the effective capacity of protein absorption and release time in a certain period.

### Morphological and phenotypical characterization of hASCs

hASCs were first morphologically characterized using inverted phase-contrast microscopy. As shown in Fig. 4b, the cells were round up when first inoculated into culture flasks, while the majority of cells were suspended in the cell culture medium. The cells adhered to the T-flask surface and exhibited fibroblast-like, spindle-shaped morphology, forming a monolayer with abundant cytoplasm and large nuclei and were in alignment [53]. For each cell, the nucleus was well stacked in the center. The cells rapidly proliferated and reached 70–80% confluence after 14 days. After 3 passages, the surface phenotype of isolated and cultured hASCs was analyzed by flow cytometry-based on the expression of *surface markers*. According to data from the flow cytometry panel (Fig.4c), isolated cells were highly positive for CD133 and CD44 (ASC surface marker) [54], while the percent of cells expressing CD34 and CD45 (both hematopoietic stem cell surface markers) was below 5%, in agreement with findings from other groups [55, 56] and confirming that hASCs possess multipotentiality and stemness features.

### hASC viability and proliferation in the PCL-P(HEMA-NIPAAm) S-2 hydrogel

An MTT assay was performed to investigate the viability of hASCs seeded on the PCL-P(HEMA-NIPAAm) hydrogel. The results show a lack of cell toxicity in hASCs plated on the PCL-P(HEMA-NIPAAm) S-2 hydrogel for 14 days, with higher viability in the presence of the hydrogel compared with the control group seeded on standard plastic culture surfaces (*p* < 0.01; Fig. 4d), suggesting that the 3D and porous structure of the scaffold had superior effects on cell survival or due to the presence of PCL in the scaffold since PCL has been reported to enhance the proliferation, adhesion, and growth of hASCs [57]. This suggests that the scaffold and its degradation products have good biocompatibility and can be considered as suitable scaffolds for cartilage tissue engineering applications.

### Chondrogenic gene expression profiles of hASCs in the PCL-P (HEMA-NIPAAm) S-2 hydrogel

Real-time RT-PCR analysis was performed to determine the ability of the PCL-P(HEMA-NIPAAm) S-2 hydrogel to support the chondrogenic differentiation of the hASCs by monitoring the expression of chondrogenic marker genes [58] such as aggrecan (major structural proteoglycan of the cartilage ECM) [59], type-II collagen (main structural component of the cartilage ECM) [60], and SOX9 (essential transcription factor for cartilage formation) [61] and of integrin β1 (most common and important receptor for collagen) [62] after 14 days. The results reveal that the expression of the four genes was changed 14 days after being cultured on the scaffold and plastic surfaces in the presence and absence of PDGF-BB (Fig. 4e). Specifically, aggrecan expression was up-regulated by PDGF-BB in cells cultured on scaffold surface or plastic substrate compared with the non-treated control group *(*62.44-fold difference, *p* < 0.0001), in agreement with the effects of this growth factor on MSC differentiation [63–65]. However, there was no significant difference in its expression between the groups [hASCs + PDGF-BB] and [hASCs + scaffold + PDGF-BB] (*p* > 0.05). Next, type-II collagen expression was significantly higher in all groups compared with the control group (up to 18.09-fold difference), although without reaching statistical significance (*p* > 0.05). Besides, maximal SOX9 expression was achieved in the group [hASCs + scaffold + PDGF-BB] (up to 48.27-fold difference, *p* < 0.0001), again concordant with the effects of PDGF on MSC chondrogenesis [63–65], while there was no significant difference between the group [hASCs + scaffold] and the control group (*p* > 0.05), suggesting that the presence of the growth factor will be most likely needed as an additional cue in the scaffold approach for optimal chondrogenesis. Finally, integrin β1 expression was induced in the groups [hASCs + PDGF-BB] and [hASCs + scaffold + PDGF-BB] compared with the control group (up to 19.55-fold difference, *p* < 0.001). Compared with the group [hASCs + scaffold], addition of PDGF-BB yielded statically significant differences (*p* < 0.01). Therefore, it could be implied that addition of certain factors increased the chondrogenic capacity of the PCL-P(HEMA-NIPAAm) hydrogel by induction of integrin β1. Although the levels of integrin β1 increased in the group [hASCs + scaffold], the changes were not statistically significant (*p* > 0.05).

## Conclusions

In this study, new thermosensitive injectable hydrogels based on polyacrylic-polyester hybrids were designed as biodegradable and biocompatible matrices for cartilage tissue engineering. The physicochemical properties of the scaffolds such as swelling ratio, hydrolytic degradation, LCST, mechanical properties, morphologies, pore size, and cytotoxicity were evaluated. The hydrogel underwent a sol-gel transition at physiological conditions (pH 7.4 and 37 °C) with typical rheological properties. According to FESEM micrographs, the PCL-P(HEMA-NIPAAm) hydrogel had a porous and nanoparticulate structure with particle size dimensions of about 62–85 nm. The biocompatibility and cell adhesion of the fabricated scaffold was evaluated by MTT assay and FESEM. These studies showed good viability and proliferation of hASCs on the scaffold. Additionally, cytocompatibility studies demonstrated that the PNIPAAm-polyester scaffold was not cytotoxic. PDGF-BB was used to induce differentiation of hASCs cultured in plastic surfaces or on the scaffold. The real-time RT-PCR results showed that hASCs cultured in the scaffold in the presence of PDGF-BB differentiated towards a chondrocyte phenotype as evidenced by chondrocyte-specific gene expression of ECM proteins such as aggrecan and type-II collagen and the cartilage-specific SOX9 transcription factor, as well as the collagen receptor integrin β1 after 14 days of culture. These results indicate that the biodegradable and biocompatible hydrogel scaffold embedded with hASCs and PDGF-BB can provide a suitable 3D environment for cell adhesion, proliferation, and differentiation of hASCs for applications in cartilage tissue engineering. This study suggests that the PCL-P(HEMA-NIPAAm) hydrogel may serve as a potential structural basis for the in vitro tissue-engineered articular cartilage construct. However, further investigations are necessary to confirm the data in different replicates in vitro. To be even more specific, the development of experimental animal models will help us to monitor the physicochemical behavior and therapeutic benefits of the PCL-P(HEMA-NIPAAm) hydrogel in in vivo conditions by injecting PCL-P(HEMA-NIPAAm) in sites of articular cartilage lesions.

## Data Availability

The datasets used and/or analyzed during the current study are available from the corresponding author on reasonable request.

## Abbreviations

2D: Two-dimensional
3D: Three-dimensional
BCA: Bicinchoninic Acid
BPO: Benzoyl peroxide
BSA: Bovine serum albumin
CP: cloud point
DMEM/LG: Low-glucose content Dulbecco’s modified Eagle medium
DMSO: Dimethyl sulfoxide
E: Elasticity
ECM: Extracellular matrix
FBS: Fetal bovine serum
GAPDH: Glyceraldehyde-3-phosphate dehydrogenase
hASCs: Human adipose-derived stem cells
HEMA: 2-*Hydroxyethyl methacrylate*
LCST: Lower critical solution temperature
MSCs: Mesenchymal stem cells
MTT: 3-(4,5-dimethylthiazol-2-yl-2,5-diphenyltetrazolium bromide
NIPAAm: N-isopropyl acrylamide
PBS: Phosphate buffered saline
PCL: Poly (ε-caprolactone)
PDGF-BB: Platelet-derived growth factor
PHEMA: Poly (2-hydroxyethyl methacrylate)
PNIPAAm: Poly N-isopropylacrylamide
Sn(Oct)_2_: Stannous octoate
WS: Working solution
ε-CL: Epsilon-caprolactone
